# DNA extraction of microbial DNA directly from infected tissue: an optimized protocol for use in nanopore sequencing

**DOI:** 10.1038/s41598-020-59957-6

**Published:** 2020-02-19

**Authors:** Karin Helmersen, Hege Vangstein Aamot

**Affiliations:** 1 Akershus University Hospital, Department of Microbiology and Infection Control, Lørenskog, 1478 Norway; 2 Akershus University Hospital and University of Oslo, Department of Clinical Molecular Biology (Epigen), Lørenskog, 1478 Norway

**Keywords:** DNA sequencing, Next-generation sequencing, Clinical microbiology

## Abstract

Identification of bacteria causing tissue infections can be comprehensive and, in the cases of non- or slow-growing bacteria, near impossible with conventional methods. Performing shotgun metagenomic sequencing on bacterial DNA extracted directly from the infected tissue may improve time to diagnosis and targeted treatment considerably. However, infected tissue consists mainly of human DNA (hDNA) which hampers bacterial identification. In this proof of concept study, we present a modified version of the Ultra-Deep Microbiome Prep kit for DNA extraction procedure, removing additional human DNA. Tissue biopsies from 3 patients with orthopedic implant-related infections containing varying degrees of *Staphylococcus aureus* were included. Subsequent DNA shotgun metagenomic sequencing using Oxford Nanopore Technologies’ (ONT) MinION platform and ONTs EPI2ME WIMP and ARMA bioinformatic workflows for microbe and antibiotic resistance genes identification, respectively. The modified DNA extraction protocol led to an additional ~10-fold reduction of human DNA while preserving *S. aureus* DNA. Including the DNA sequencing and bioinformatics analyses, the presented protocol has the potential of identifying the infection-causing pathogen in infected tissue within 7 hours after biopsy. However, due to low number of *S. aureus* reads, positive identification of antibiotic resistance genes was not possible.

## Introduction

As next-generation sequencing (NGS), with its multitude of advantages, is approaching acceptance as the gold standard in bacteriology^1^, the demand for optimal DNA extraction procedures are increasing. Being able to extract microbial DNA directly from human samples followed by shotgun metagenomic sequencing, where all DNA in a complex sample is identified, can reduce time to diagnosis and targeted treatment. This is especially important in cases with slow-growing or difficult to cultivate microbes. However, in most types of human samples, the proportion of human DNA (hDNA) to microbial DNA is overwhelming and reduces the probability of identifying microbes using NGS technologies. Therefore, a DNA extraction protocol capable of depleting hDNA while simultaneously preserving microbial DNA is fundamental to the improvement of NGS microbe detection.

The Ultra-Deep Microbiome Prep kit (Molzym, Bremen, Germany) is a DNA extraction kit that combines removal of host DNA and extraction of enriched microbial DNA from a variety of sample types, including biopsies. It enables detection and identification of viable bacteria and fungi including the nonculturable. The kit has been used for shotgun metagenomic sequencing of broncho-alveolar lavage fluid using Illumina sequencing platform^2^. Additional examples are found in the microbiota characterization of human breast tissue biopsies, as well as the profiling of oral bacteria on pathologically changed heart valves using 16 S rRNA sequencing on an Ion PGM Sequencer (Life Technologies) and Sanger sequencing, respectively^3,4^. However, the NGS sequencing platforms widely used for metagenomic sequencing, such as Ion Torrent and Illumina, require comprehensive pre-sequencing preparation of samples and require the sequencing run to completion before analysis can start (the exception being a recently described method that can analyze raw Illumina data before run completion (LiveKraken^5^). These obstacles may be overcome by using nanopore sequencing technology (Oxford Nanopore Technologies (ONT), Oxford, UK) where pre-sequencing preparation is short (15 min–2 h depending on DNA concentration) and data analysis can be done in near real-time using the web-based EPI2ME bioinformatics analysis platform including WIMP (What’s In My Pot)^6^ for microbe identification, and ARMA (Antibiotic Resistance Mapping Application) for antibiotic resistance genes based on the Comprehensive Antibiotic Resistance Database (CARD, http://arpcard.mcmaster.ca/).

Patients with orthopedic implant-associated infections (OIAI) could benefit from these technological advances. Although these infections are infrequent per se, with an overall surgical site infection rate following implant surgery of 3%^7^, the total number of patients undergoing orthopedic implant surgery is high and increasing. The implications of these infections are severe and relying on empirical antibiotic therapy may cause suboptimal or inefficient treatment, increasing risk of poor functional outcome and mortality. The expeditious identification of these infective agents is of major importance for patient treatment and could improve outcome.

Conventional microbiological diagnostics of OIAI require that 5 biopsies from each patient be cultivated on several different media for at least 5 days^8^. An additional 24 hours is needed for determination of phenotypic antibiotic susceptibility.

In this proof of concept study, the aim was to demonstrate the feasibility of using a modified version of the Ultra-Deep Microbiome Prep kit for DNA extraction and subsequent shotgun metagenomic sequencing using ONT’s nanopore sequencing and bioinformatic platform for near real-time identification of microbes and antibiotic resistance genes directly from infected tissue.

## Material and Methods

Diagnostic soft tissue biopsies were taken from patients suffering orthopedic-implant associated infections (OIAI) at Akershus University Hospital from January 2017 to December 2018. The biopsies were taken from areas directly adjacent to the infected implant. The patients met the criteria for an orthopedic implant-associated infection as described by Parvizi^9^.

Each biopsy was divided in 2, where 1 piece was cultivated following conventional microbiological diagnostics and the other available for sequencing was initially frozen at −80 °C. Antibiotic susceptibility testing was performed according to the guideline from the European Committee on Antimicrobial Susceptibility Testing^10^ and EUCAST breakpoints were utilized to categorize the isolate as sensitive (S), intermediate (I) or resistant (R)^11^. A total of 33 patient were included. From this collection, biopsies from 3 patients (a total of 13 unique biopsies) were selected based on the semi-quantification of *Staphylococcus aureus* growth during routine diagnostic cultivation (ranging from broth-only to dense). Patients with *S. aureus* infection were selected in this proof of concept study as *S. aureus* is among the most common cause of OIAI^12^.

The Regional Committee for Medical and Health Research Ethics (South-East, 2016/1929) and the Akershus University Hospital’s local Data Protection Officer (17/024) approved this study. The patients gave their written, informed consent. All research was performed in accordance with relevant guidelines/regulations.

### DNA extraction

Based on preliminary results from the master’s thesis “Rapid molecular diagnostic tool for identification of bacteria causing orthopedic implant-related infections” (unpublished), the original protocol for the Ultra-Deep Microbiome Prep kit for DNA extraction could benefit from optimization of the hDNA depletion step. Therefore, the present modified protocol with additional cell lysis and human DNA depletion steps were tested.

**Figure 1:**
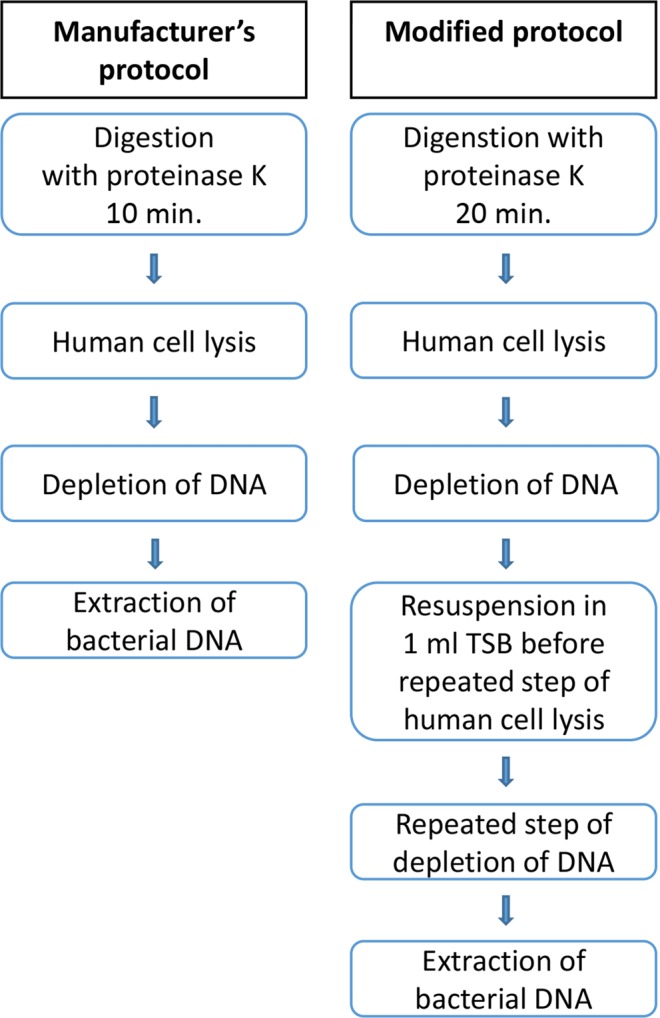
Manufacturer’s and modified protocol of bacterial DNA extraction from human biopsies using Ultra-Deep Microbiome Prep kit (Molzym, Bremen, Germany).

Each of the 13 included biopsies were divided into two approximately equally sized pieces (P I and P II) and weighed (Table 1) before carrying out DNA extraction using the Ultra-Deep Microbiome Prep kit. Handling of the biopsies and DNA extraction were performed in a type 2 microbiological safety cabinet. Biopsy P I was extracted using the manufacturer’s protocol. For biopsy P II, DNA was extracted using the following modifications; prolongation of the first incubation with proteinase K from 10 to 20 min., followed by the lysis of human cells and degradation of extracellular DNA. Pellet resuspension in 1 mL TSB followed, and the lysis of human cells and degradation of extracellular DNA step was repeated (Fig. 1). The centrifugations were performed at 14 000 × g in both protocols.

**Table 1:** Description of biopsies and semi-quantification of *S. aureus* culturing.

Patient - Biopsy number	Culture growth of *S. aureus*	Weight (grams) P I	Weight (grams) P II
1 - Biopsy 1	Dense	0.040	0.037
1 - Biopsy 2	Dense	0.091	0.082
1 - Biopsy 3	Moderate	0.042	0.048
1 - Biopsy 4	Dense	0.040	0.053
1 - Biopsy 5	Sparse	0.040	0.034
2 - Biopsy 1	Sparse	0.032	0.043
2 - Biopsy 2	Sparse	0.066	0.052
2 - Biopsy 3	Single colonies	0.020	0.024
2 - Biopsy 4	Sparse	0.052	0.051
2 - Biopsy 5	Moderate	0.021	0.022
3 - Biopsy 1	Broth only	0.037	0.040
3 - Biopsy 2	Culture negative	0.071	0.084
3 - Biopsy 3	Culture negative	0.006	0.012

### Assessment of optimization steps (qPCR)

To compare and assess the effect of the optimization steps, two qPCRs were performed for the detection of human DNA (human β-globin gene *HBB*) and *S. aureus* DNA (*nuc*-gene).

A previously published protocol was used for detection of *HBB*^13^. The *nuc* qPCR consisted of a 20 µl reaction containing primers and probe sequences and concentrations from Tunsjø and co-workers^14^, and TaqMan FAST Universal PCR Mastermix 2X (Applied Biosystems, Thermo Fisher Scientific, Waltham, MA, USA). Amplification conditions for the *nuc* qPCR were as follows: 95 °C for 20 sec, 35 cycles of 95 °C for 1 sec and 60 °C for 20 sec.

Both qPCR protocols were performed on a 7900HT Fast Real-time PCR instrument (Applied Biosystems). All samples were run in parallel and DNA concentrations were estimated using 10-fold dilution standard curves for both qPCR assays. Samples with Ct-values higher than 40 were characterized as negative and, in calculation of the mean Ct-values, calculated as 40.

### Library preparation and MinION sequencing

Library preparation was performed using Rapid PCR Barcoding Kit (SQK-RPB004, Oxford Nanopore Technologies) following manufacturers guidelines (RPB_9059_v1_revD_08Mar2018). The input volume of all samples was 3 µl and to best preserve all DNA, the pooling of samples was performed without quantification after the AMPure XP beads step. 1 µl of RAP (Rapid Adapter) was added to 10 µl of the pooled eluate and the library was kept on ice until loaded onto the flow cell. One library was prepared for each patient, including five P I samples and five P II samples, indexed and multiplexed on one flow cell. One no template control (NTC) was included in the library preparation for patients 2 and 3.

Shotgun metagenomic sequencing was carried out on a MinION sequencer (Oxford Nanopore Technologies) using R9.4.1 FLO-MIN 106 flow cells. The operating software MinKNOW was used for local base calling (Patient 1: MinKNOW v. 1.15.4, Patients 2 and 3: MinKNOW v. 3.3.2, Guppy 3.0.3). Demultiplexing and identification of both pathogen and antibiotic resistance genes were performed using the cloud-based bioinformatics platform, EPI2ME (Patient 1: EPI2ME v. 2.57.1769546, Patients 2 and 3: EPI2ME v 2.59.1896509). The QC and Barcoding, WIMP, and ARMA workflows were employed using default Q-score ≥7. The MinION was run for up to 48 hours. The results files from the 3 workflows were combined in order to extract the run data for each read. The protein homolog model of antibiotic resistance genes with average alignment accuracy of ≥90% were reported.

## Results

Descriptions of the biopsies and results from cultivation are presented in Table 1. The results of the qPCR are presented in Table 2. For detailed results from MinION sequencing, see Supplement File 1. The modified protocol increased the DNA extraction procedure to ~3 hours and the MinION library preparation took ~3 hours, resulting in ~6 hours sample preparation before sequencing.

**Table 2:** Results of qPCR quantification of human and *S. aureus* DNA after DNA extraction of *S. aureus* infected biopsies following manufacturer’s (P I) and modified protocol (P II).

Patient - Biopsy number - Protocol	Mean Ct-values of replicates		Concentration (ng/µl)	
	Human *β-globin* gene	*S. aureus* *nuc*-gene	*β-globin*	*nuc*
1 - Biopsy 1 PI	29.1	26.7	7.85	0.043
1 - Biopsy 1 PII	31.8	27.1	0.27	0.033
1 - Biopsy 2 PI	27.8	28.1	4.10	0.018
1 - Biopsy 2 PII	31.1	28.9	0.42	0.010
1 - Biopsy 3 PI	31.3	31.9	0.37	0.0016
1 - Biopsy 3 PII	35.1	30.8	0.027	0.003
1 - Biopsy 4 PI	25.7	27.4	17.50	0.020
1 - Biopsy 4 PII	29.9	26.4	1.00	0.050
1 - Biopsy 5 PI	29.3	28.5	1.50	0.010
1 - Biopsy 5 PII	32.8	30.3	0.14	0.0040
2 - Biopsy 1 PI	34.1	Negative	0.055	0
2 - Biopsy 1 PII	37.6	Negative	0.0050	0
2 - Biopsy 2 PI	33.9	36.5	0.060	9.3 e-5
2 - Biopsy 2 PII	Negative	Negative	0	0
2 - Biopsy 3 PI	Negative	Negative	0	0
2 - Biopsy 3 PII	Negative	Negative	0	0
2 - Biopsy 4 PI	38.0	Negative	0.0030	0
2 - Biopsy 4 PII	Negative	Negative	0	0
2 - Biopsy 5 PI	27.1	38.6	0.0030	2.7 e-5
2 - Biopsy 5 PII	Negative	Negative	0	0
3 - Biopsy 1 PI	38.8	Negative	0.0020	0
3 - Biopsy 1 PII	36.8	Negative	0.0090	0
3 - Biopsy 2 PI	31.0	35.1	0.46	2.2 e-4
3 - Biopsy 2 PII	34.0	33.8	0.050	5.1 e-4
3 - Biopsy 3 PI	31.2	35.4	0.40	1.8 e-4
3 - Biopsy 3 PII	Negative	34.3	0	3.7 e-4

### Patient 1

Patient 1 was chosen based on the dense growth of *S. aureus* by standard cultivation of the biopsies (Table 1). The human *β-globin* gene qPCR showed an increase in mean Ct-values from biopsies using manufacturer’s protocol, P I, 28.6 [25.7–31.3] to 32.1 [29.9–35.1] (3.5 Ct-values) using modified protocol, P II, corresponding to approximately a 10-fold reduction of human DNA using the modified protocol (P II). For the related *nuc* qPCR, Ct-values stayed virtually unchanged resulting in mean Ct-values of 28.5 [26.7–31.9] for P I, and 28.7 [26.4–30.8] for P II.

MinION sequencing stopped after 17 hours and showed a reduction of total number of hDNA reads in the 5 biopsies from 60063 using PI to 1755 reads using PII. *S. aureus* reads increased from 613 reads (PI) to 3831 reads (PII). This corresponds to roughly a 34-fold reduction of human DNA reads, and a 6-fold increase in *S. aureus* reads. The number of reads identified by WIMP as microbes other than *S. aureus* increased from 142 reads to 271 reads from PI to PII, respectively. In P I, these reads consisted mainly of *S. aureus* subspecies (N = 34) and *Malassezia globosa* (N = 12), whereas using P II the majority consisted of *S. aureus* subspecies reads (N = 188), staphylococcus phages (N = 20) or staphylococcus viruses (N = 17). Unfortunately, no controls were included in this run. All biopsies were positive for *S. aureus* during the first hour of sequencing (Supplement File 1), so identification of the infection-causing pathogen would have been possible within 7 hours after biopsy.

Phenotypic antibiotic resistance testing showed resistance to ciprofloxacin. Using the ARMA bioinformatic tool for identification of antibiotic resistance genes, 4 alignments of *tetC* (average accuracy 91.0%), 3 alignments of *arlS* (average accuracy 91.3%) and 1 alignment of sav1866 (average accuracy 90.0%) were identified in the biopsies extracted by the original protocol. Using the modified DNA extraction protocol, 8 alignments of *arlS* (average accuracy 91.9%) and 7 alignments of *sav1866* (average accuracy 91.3%) were identified. *arlS* is part of arlRS that regulates *norA* and thereby ciprofloxacin resistance and corresponds to the phenotypically detected antibiotic resistance.

### Patient 2

Cultures from patient 2 produced intermediate growth of *S. aureus* in all 5 biopsies (Table 1). The qPCR showed a reduction of human *β-globin* in all P II biopsies compared to P I biopsies with a mean Ct-value of 34.6 [27.1–40.0] in P I biopsies and a mean Ct-value of 39.5 [37.6–40.0] in P II biopsies (4.8 Ct-values). This corresponds to more than a 50-fold reduction in hDNA. *nuc* was only detected in Biopsy 2 and 5 following manufacturer’s protocol, whereas all biopsies extracted according to modified protocol were negative for *nuc*.

MinION sequencing stopped at 48 hours. The total human sequencing reads in biopsies from Patient 2 were reduced from 856 275 reads (P I) to 1181 (P II), whereas the total number of *S. aureus* reads were reduced from 637 to 155, respectively. The reduction of *S. aureus* reads was mainly due to biopsy 3 that showed a significant reduction of reads from PI to PII (565 reads to 26 reads in total, Supplement 1). This difference in sensitivity could potentially affect interpretation which requires a minimum of 2 of 5 biopsies to be positive for a pathogen to be deemed to be the causative agent.

Reads identified as other microbes by WIMP, were reduced from 2376 reads (P I) to 48 reads (P II). The majority of the other microbes reads were identified as *Malassezia globosa* (N = 689 reads) and Opisthokonta (N = 512) in P I biopsies and *S. aureus* subspecies (N = 27) in P II biopsies. The NTC had a total of 21 reads of which 20 were human and 1 was *Malassezia globosa*. Due to few *S. aureus* reads, at least 4 hours of sequencing would have been required to positively identify the causative agent, extending the total time from biopsy to pathogen ID to 10 hours.

Phenotypic antibiotic resistance testing showed resistance to ciprofloxacin and penicillin. Biopsies extracted with manufacturer’s protocol showed 1 alignment with *norA* (average alignment accuracy 93.0%) using ARMA, whereas biopsies extracted with modified protocol showed 1 alignment with *mepA* (average alignment accuracy 94.0%). *norA* confers resistance to ciprofloxacin, whereas *mepA* confers resistance to tetracyclins. No antibiotic resistance genes alignments were identified in the NTC.

### Patient 3

Patient 3 cultures had growth of *S. aureus* in 2 of 4 biopsies only after pre-cultivation in broth. Of these 4 biopsies, 3 were available for DNA extraction (Table 1). The mean Ct-values for *β-*globin increased from 33.7 [31.0–38.8] in P I to 36.9 [34.0–40.0] in P II samples, whereas the mean Ct-values for *nuc* were stable with 36.8 [35.1–40.0] for P I and 36.0 [33.8–40.0] for P II, respectively. This corresponds to a ~10-fold decrease of human DNA.

MinION sequencing stopped after 48 hours and the total number of human DNA reads were reduced from 3 697 756 in P I biopsies to 8280 in P II biopsies. Total *S. aureus* DNA reads increased from 24 to 215 reads (Supplement File 1). The total number of reads for other microbes were reduced from 6058 (P I) to 112 reads (P II). The majority of other microbes in the P I biopsies consisted of *Malassezia globosa* (N = 4067 reads), and *S. aureus* subspecies (N = 88 reads) in P II biopsies. Using P II, *S. aureus* was identified in the first hour of sequencing, whereas using P I, *S. aureus* was identified during the fourth hour of sequencing. In P II, only 1 *S. aureus* read was identified in biopsy 1 (positive after pre-cultivating in broth), whereas the 2 culture-negative biopsies showed *S. aureus* reads from the first hour of sequencing. The NTC had a total of 11 (all human) reads. *S. aureus* was identified within the first hour of sequencing, resulting in a total time from biopsy to pathogen ID of 7 hours.

Phenotypic antibiotic resistance testing showed resistance to penicillin. No resistance genes were detected in the biopsies extracted with the manufacturer’s protocol and 2 alignments of *mepA* (average alignment accuracy 90.0%) were identified in the biopsies extracted with the modified protocol and using the ARMA pipeline. The NTC displayed no alignments.

## Discussion

A modified version of the Ultra-Deep Microbiome Prep kit for DNA extraction, facilitated a further ~10-fold reduction of human DNA while preserving *S. aureus* DNA. Consequently, this resulted in increased sensitivity of shotgun metagenomic sequencing and improved pathogen identification. This modified protocol has the potential of identifying the infection-causing pathogen in infected tissue in as little as 7 hours after biopsy. However, due to low number of *S. aureus* reads, positive identification of antibiotic resistance genes was not possible.

### Reduction of human DNA

Culture-independent diagnostic of infections has the potential to improve time to pathogen identification drastically as many pathogens can be difficult to cultivate or they grow slowly. When using shotgun metagenomic sequencing, hDNA poses challenges when the human to microbe ratio is high, as it is in tissue. The challenge is even greater in tissue from implant-related infections as implants has been shown to reduce the number of microbes needed to cause an infection. An animal study using *S. aureus* as study organism showed that implants reduces the required number of microbes to establish an infection from 10^8^ colonizing forming units (cfu) to a few as 100 cfu^15^. Removing hDNA during the DNA extraction procedure instead of removing human reads after sequencing has obvious advantages as the sensitivity of the DNA sequencing will increase as the human to pathogen DNA ratio is reduced. However, removing human DNA during DNA extraction also results in low DNA concentration in the resulting eluate. We were thus required to use the Rapid PCR Barcoding Kit. This kit allows for a low amount of input DNA, but as a consequence of the PCR step, library preparation time increases accordingly. Despite this increase in preparation time, the protocol still permits same-day diagnostics of implant associated infections.

### Identification of pathogens

The 3 patients with OIAI caused by *S. aureus* were selected because they were infected with one of the most common pathogen causing these infections^12^. They were also showing variable amount of *S. aureus* by standard cultivation varying from only positive after pre-cultivation in broth to dense growth, even two biopsies in Patient 3 were negative.

Shotgun metagenomic sequencing was able to identify *S. aureus* in all biopsies, using EPI2ME’s WIMP workflow. However, the percentage of *S. aureus* reads were improved using the modified DNA extraction protocol (Supplement File 1). Overall, this indicates similar specificity as culturing and a higher sensitivity for the modified protocol compared to the manufacturer’s protocol.

Results of *β-globin* qPCR indicated a considerable reduction of hDNA when comparing manufacturer’s to modified protocol. This reduction could explain the increase in *S. aureus* reads as more of the sequencing capacity would be available for *S. aureus* DNA. Noteworthy, biopsy 1 in Patient 3 was positive for *S. aureus* after pre-cultivation in broth and had only one read identified as *S. aureus*, whereas the other two biopsies from this patient were culture-negative, but sequencing identified *S. aureus* within the first hour. In this case, sequencing showed a higher sensitivity than conventional culturing.

When investigating individual biopsies, *nuc* qPCR showed both increase and decrease in concentration when comparing manufacturer’s and modified protocol (Table 2). This may be explained by the tissue composition of the biopsies (fat, muscle, connective tissue etc) and that performing the additional lysis and degradations steps in the modified protocol may affect the extraction of *S. aureus* DNA differently in different tissue compositions.

Other microbes were also identified by WIMP. They were, however, less salient, and in some cases completely eliminated, using the modified protocol. None would have led to a challenging interpretation as they were not considered plausible causes of an OIAI. When sequencing biopsies from Patients 2 and 3, a NTC were included. They did not reveal contaminants that could affect interpretation.

Publications on shotgun metagenomics sequencing of implant-related infected tissue without prior cultivation are scarce^16^. Those which are related to orthopedic implant infections are usually performed on other types of patient samples like sonication fluid^17,18^ or analyzed by multiplex PCR which is inherently limited by its targeted approach^19^.

### Identification of resistance genes

Although the phenotypic analyses showed that *S. aureus* from the 3 included patients expressed different antibiotic resistance, these could only be detected sufficiently using the modified DNA extraction protocol. Patient 1’s ciprofloxacin resistance could be explained by the presence of *arlS*, but an additional resistance marker was identified by sequencing, *sav1866. sav1866* is in the multidrug ABC transporter family and it facilitates the export of diverse cytotoxic drugs across cell membranes^20^. The highest total number of *S. aureus* reads in this study was 3831 (Patient 1, P II). Given that the expected read length using the Rapid PCR Barcoding kit is ~3000 base pairs, the estimated coverage of a *S. aureus* genome (≈3 MB) would be less than 4X. In order to ensure identification of antibiotic resistance by sequencing, a higher load of bacteria is needed. This is often not possible without cultivation.

This study has limitations. As a proof of concept study, biopsies from only 3 patients were included. However, these biopsies were chosen to test the protocol on different degrees of *S. aureus* growth. Negative controls for DNA extraction should have been included in order to adjust for possible contamination from the extraction kit reagents. These will be included in future studies. Additionally, all biopsies were stored at −80 °C before DNA extraction. This can cause unwanted bacterial cell lysis, leading to loss of bacterial DNA during the hDNA degradation steps. As the sample preparation kit includes a PCR step, the amplification of the DNA may be biased towards shorter DNA fragments and when sequenced may not represent the “true” composition of the biopsy. Finally, the modified protocol needs to be tested and verified on larger number of biopsies and other pathogens.

In conclusion, the modified protocol with additional removal of human DNA, is promising for same-day identification of pathogens directly from infected tissue using Oxford Nanopore’s shotgun metagenomic sequencing platform with WIMP and ARMA bioinformatics workflows. The identification of antibiotic resistance, however, may be challenging due to the inherently low concentration of pathogens in tissue biopsies. Further investigations in a larger cohort will be performed.

## Supplementary information

Supplemental information 1.
