# Supplementary material for: DNA extraction of microbial DNA directly from infected tissue: an optimized protocol for use in nanopore sequencing
Source: Sci Rep. 2020 Feb 19;10:2985. doi: 10.1038/s41598-020-59957-6 (PMC7031281; doi:10.1038/s41598-020-59957-6)
Supplement: Supplementary file 1 — Supplemental information 1. [file 41598_2020_59957_MOESM1_ESM.pdf]

DNA extraction of microbial DNA directly from infected tissue: an optimized protocol for use in nanopore sequencing

Karin Helmersen, Hege Vangstein Aamot

Patient 1

Protocol	Taxonomic ID	Biopsy	1	2	3	4	5	6	7	8	9	10	11	12	13	14	15	16	17	Total
			Start time base calling in hours																	
Manufacturer's protocol	Human	B1	284	79	16	6	9	3	1	1	1	1	0	0	0	0	0	0	0	401
		B2	33598	9012	2099	1284	983	453	229	127	65	47	34	17	10	4	2	16	3	47983
		B3	2342	623	147	85	64	25	19	3	8	7	1	1	0	0	0	0	0	3325
		B4	1217	321	81	47	37	16	11	6	4	1	0	0	0	0	0	0	0	1741
		B5	4654	1217	300	176	138	46	30	20	11	11	6	3	1	0	0	0	0	6613
		Total	42095	11252	2643	1598	1231	543	290	157	89	67	41	21	11	4	2	16	3	60063
Modified protocol	Human	B1	6	2	0	0	0	0	0	0	0	0	0	0	0	0	0	0	0	8
		B2	215	53	18	9	9	2	1	1	0	1	0	0	0	0	0	1	0	310
		B3	777	192	39	29	20	15	6	3	1	0	0	1	0	0	1	1	0	1085
		B4	12	3	1	0	1	0	0	0	0	0	0	0	0	0	0	0	0	17
		B5	232	65	16	11	9	2	0	0	0	0	0	0	0	0	0	0	0	335
		Total	1242	315	74	49	39	19	7	4	1	1	0	1	0	0	1	2	0	1755
Manufacturer's protocol	*S. aureus*	B1	4	2	0	0	0	0	1	1	0	0	0	0	0	0	0	0	0	8
		B2	330	82	21	8	8	1	2	6	0	0	0	0	0	0	0	1	0	459
		B3	32	13	3	2	2	0	0	1	0	0	0	0	0	0	0	1	0	54
		B4	30	10	3	2	0	0	0	0	0	0	0	0	0	0	0	0	0	45
		B5	31	7	4	0	2	1	0	1	0	1	0	0	0	0	0	0	0	47
		Total	427	114	31	12	12	2	3	9	0	1	0	0	0	0	0	2	0	613
Modified protocol	*S. aureus*	B1	3	1	0	0	0	0	0	0	0	0	0	0	0	0	0	0	0	4
		B2	646	125	43	21	21	12	4	4	0	1	0	1	2	0	0	0	0	880
		B3	1493	411	94	57	54	22	10	6	5	1	1	1	1	0	0	0	0	2156
		B4	67	12	5	2	5	1	0	1	0	0	0	0	0	0	0	0	0	93
		B5	479	125	26	26	22	9	4	2	2	2	1	0	0	0	0	0	0	698
		Total	2688	674	168	106	102	44	18	13	7	4	2	2	3	0	0	0	0	3831
Manufacturer's protocol	Others	B1	3	1	0	0	0	1	0	0	0	0	0	0	0	0	0	0	0	5
		B2	63	25	3	6	4	2	0	0	0	0	1	0	0	0	0	0	0	104
		B3	6	1	0	0	1	0	0	0	0	0	0	0	0	0	0	0	0	8
		B4	5	0	0	0	0	0	0	0	0	0	0	0	0	0	0	0	0	5
		B5	13	5	2	0	0	0	0	0	0	0	0	0	0	0	0	0	0	20
		Total	90	32	5	6	5	3	0	0	0	0	1	0	0	0	0	0	0	142
Modified protocol	Others	B1	0	0	1	0	1	0	0	0	0	0	0	0	0	0	0	0	0	2
		B2	39	11	5	0	3	1	0	0	0	1	0	0	0	0	0	0	0	60
		B3	98	29	9	6	1	0	1	1	0	0	0	0	0	0	0	0	0	145
		B4	8	3	0	0	0	0	0	0	0	0	0	0	0	0	0	0	0	11
		B5	40	10	0	1	2	0	0	0	0	0	0	0	0	0	0	0	0	53
		Total	185	53	15	7	7	1	1	1	0	1	0	0	0	0	0	0	0	271

Patient 2

Protocol	Taxonomic ID	Biopsy	1	2	3	4	5	6	7	8	9	10	11	12	13	14	15	16	17	18	19	20	21	22	23	24	25	26	27	28	29	30	31	32	33	34	35	36	37	38	39	40	41	42	43	44	45	46	47	48	Total
			Start time base calling in hours																																														
Manufacturer's protocol	Human	B1	24232	20434	17705	13670	18124	13684	13721	14655	12478	14726	11096	14045	10291	12820	10403	10414	11173	25760	33330	31713	31991	29810	26703	27676	26638	24921	23722	21921	21005	20860	18973	17513	16887	14645	13259	12750	11227	9583	8394	6730	6319	5215	4230	4169	3322	2496	2254	1679	749366
		B2	2208	1847	1609	1219	1590	1183	1160	1286	1053	1326	1001	1224	955	1097	920	943	986	2175	2829	2899	2797	2634	2313	2429	2353	2171	2138	2007	1880	1895	1705	1618	1518	1337	1135	1163	967	892	810	637	591	500	406	396	321	262	226	156	66767
		B3	1265	1092	946	716	901	722	659	690	618	767	583	683	478	634	507	544	555	1229	1609	1598	1557	1454	1281	1413	1277	1268	1186	1052	1052	1064	963	909	866	756	717	655	575	477	438	315	317	271	247	191	180	140	133	82	37632
		B4	8	10	10	4	5	4	4	4	4	6	5	5	4	8	3	4	3	4	8	9	18	8	5	11	9	13	5	7	5	6	3	2	3	8	1	2	1	3	3	2	1	0	0	2	0	0	0	0	230
		B5	78	70	65	41	55	41	37	39	41	47	44	36	27	48	24	33	25	80	93	87	118	88	83	70	75	80	79	56	57	58	79	56	56	36	34	45	42	34	24	23	16	15	12	11	6	6	7	3	2280
		Total	27791	23453	20335	15650	20675	15634	15581	16674	14194	16872	12729	15993	11755	14607	11857	11938	12742	29248	37869	36306	36481	33994	30385	31599	30352	28453	27130	25043	23999	23883	21723	20098	19330	16782	15146	14615	12812	10989	9669	7707	7244	6001	4895	4769	3829	2904	2620	1920	856275
Modified protocol	Human	B1	0	2	5	1	2	2	4	2	3	1	2	2	1	0	4	1	1	1	7	0	1	3	2	1	4	0	2	3	4	0	2	2	2	1	2	0	1	1	0	1	0	0	0	0	1	0	1	0	75
		B2	33	29	35	8	21	12	16	14	19	10	17	15	8	18	12	7	10	34	40	32	34	33	37	33	26	30	18	18	22	22	18	20	14	10	10	14	15	6	7	11	6	3	3	4	5	4	3	1	817
		B3	6	6	4	4	6	3	4	1	3	5	3	6	1	3	2	4	6	7	11	5	10	4	7	9	6	12	5	11	3	5	2	9	1	3	2	1	4	5	4	1	3	0	0	2	3	3	0	0	205
		B4	2	1	0	1	3	0	1	1	2	0	0	2	1	0	1	0	2	1	2	3	3	1	0	1	2	0	5	2	0	2	0	1	0	2	1	3	0	2	2	0	0	0	0	0	1	0	0	0	51
		B5	3	6	0	2	0	0	0	1	0	3	0	0	2	1	1	1	0	1	0	0	0	0	0	4	2	2	1	0	0	1	0	0	1	0	0	0	0	0	0	0	0	0	1	0	0	0	0	0	33
		Total	44	44	44	16	32	17	25	19	27	19	22	25	13	22	20	13	19	44	60	40	48	41	46	48	40	44	31	34	29	30	22	32	18	16	15	18	20	14	13	13	9	3	4	6	10	7	4	1	1181
Manufacturer's protocol	*S. aureus*	B1	0	0	0	0	0	0	2	1	0	0	0	0	1	2	1	0	0	0	0	2	4	2	5	0	0	1	1	2	0	0	3	1	0	1	0	0	0	0	0	0	0	1	0	0	1	0	0		31
		B2	0	0	0	1	1	3	0	0	0	0	0	0	0	0	0	1	1	1	1	1	0	1	0	1	1	0	2	0	1	0	1	0	0	1	0	0	1	0	1	0	0	0	0	0	0	1	0		21
		B3	12	14	7	12	12	10	8	9	12	11	5	17	6	10	9	8	12	24	31	16	24	18	20	24	19	27	16	22	14	20	17	11	10	8	10	6	12	5	7	3	9	6	2	4	4	0	2		565
		B4	0	0	0	0	0	0	0	1	0	0	1	0	0	0	1	0	0	1	0	0	0	0	0	0	1	0	0	0	0	0	1	0	0	0	0	0	0	0	0	0	0	0	0	0	0	0	0		6
		B5	0	1	0	1	1	0	0	0	1	0	1	0	0	0	0	0	0	0	0	0	1	0	0	1	1	1	1	0	0	0	0	1	0	0	0	2	1	0	0	0	0	0	0	0	0	0	0		14
		Total	12	15	7	14	14	13	10	11	13	11	7	17	7	12	11	9	13	26	32	19	29	21	25	26	22	29	20	24	15	20	22	13	10	10	10	8	14	5	8	3	9	7	2	4	5	1	2		637
Modified protocol	*S. aureus*	B1	1	1	2	4	0	2	0	3	3	2		1	0	1	1		3	2	0	2	2	1	4	6	0	0	0	1	2	1	1	3	2	3	1	1	0	0	1	0		0	0	0					57
		B2	0	0	0	0	0	0	0	0	0	0		0	0	0	0		0	0	0	0	0	0	0	0	0	1	0	0	0	0	0	0	0	0	0	0	0	1	0	0		0	0	0					2
		B3	1	1	0	0	1	0	0	0	2	0		1	0	2	0		0	1	2	1	1	1	0	0	0	2	0	2	0	0	2	1	1	1	0	0	0	1	0	0		2	0	0					26
		B4	0	0	0	0	0	0	0	0	0	0		0	0	0	1		0	0	0	0	0	2	0	0	0	0	0	0	0	0	0	0	0	0	0	0	0	0	0	0		0	0	0					3
		B5	3	3	1	1	1	3	2	0	0	0		3	1	2	1		0	3	2	2	4	4	5	0	3	1	1	0	0	2	2	1	3	2	2	2	1	2	1	1		0	1	1					67
		Total	5	5	3	5	2	5	2	3	5	2		5	1	5	3		3	6	4	5	7	8	9	6	3	4	1	3	2	3	5	5	6	6	3	3	1	4	2	1		2	1	1					155
Manufacturer's protocol	Others	B1	29	26	34	29	26	23	23	25	20	14	16	23	15	21	18	25	18	40	57	61	52	69	51	56	61	54	61	40	44	55	53	69	72	49	53	54	50	42	47	41	38	52	32	40	38	22	19	14	1871
		B2	7	4	6	2	7	4	2	3	4	4	2	3	2	3	1	3	0	6	6	8	8	16	5	4	7	10	4	6	2	9	9	4	5	3	5	5	9	4	4	8	3	3	4	3	5	0	4	2	228
		B3	8	3	8	4	6	6	7	4	2	5	7	1	1	4	5	2	2	6	15	7	9	8	6	7	11	7	7	8	8	12	10	7	8	4	4	5	5	7	6	3	2	4	4	2	0	0	1	0	258
		B4	0	0	1	0	0	1	1	0	0	0	0	0	1	0	0	0	1	0	0	0	0	0	1	1	0	0	0	0	1	0	0	0	1	0	0	0	0	0	0	1	1	0	0	0	0	0	0	0	11
		B5	0	0	0	0	1	0	1	0	0	1	0	0	0	0	0	0	0	0	0	1	0	0	0	3	0	0	0	0	1	0	0	0	0	0	0	0	0	0	0	0	0	0	0	0	0	0	0	0	8
		Total	44	33	49	35	40	34	34	32	26	24	25	27	19	28	24	30	21	52	78	77	69	93	63	71	79	71	72	54	56	76	72	80	86	56	62	64	64	53	57	53	44	59	40	45	43	22	24	16	2376
Modified protocol	Others*	B1	0		0		0	1		1	0	0		0	0				0	0	1		1		0	0	1		0	2	1	0	1	0		0		0				0	1	0							10
		B2	0		1		1	0		0	0	0		0	0				0	1	1		0		0	1	1		0	0	0	0	0	0		0		0				1	0	0							7
		B3	1		0		0	1		0	1	1		0	0				1	0	1		1		2	1	1		1	0	0	1	0	0		0		1				0	0	1							15
		B5	0		0		0	0		0	1	1		1	1				0	0	1		1		2	1	2		1	0	0	1	0	1		1		0				0	1	0							16
		Total	1		1		1	2		1	2	2		1	1				1	1	4		3		4	3	5		2	2	1	2	1	1		1		1				1	2	1							48
		* No reads registered on biopsy 4																																															
NTC	Human		1	2	1				1		0		1				2	1	1	1	1		1	2	2	1															1	1									20
	Others		0	0	0				0		1		0				0	0	0	0	0		0	0	0	0															0	0									1
		Total	1	2	1				1		1		1				2	1	1	1	1		1	2	2	1															1	1									21

Patient 3

Protocol	Taxonomic ID	Biopsy	0	1	2	3	4	5	6	7	8	9	10	11	12	13	14	15	16	17	18	19	20	21	22	23	24	25	26	27	28	29	30	31	32	33	34	35	36	37	38	39	40	41	42	43	44	45	46	47	48	Total
			Start time base calling in hours																																															
Manufacturer's protocol	Human	B1	22011	71803	72826	92553	98918	94128	93677	94809	91359	91759	90780	89687	87369	85967	81553	78852	80024	78598	74514	73398	73490	72399	69331	65087	64675	62146	62469	61789	56344	56175	56742	52079	50021	50321	45478	41978	40564	38166	37659	38004	37908	34300	32718	30023	27051	30428	27729	23524	13317	2996500
		B2	1723	5375	5335	6807	7266	6875	6929	6890	6604	6643	6757	6433	6497	6353	5902	5718	5893	5827	5576	5372	5605	5377	5227	4889	4847	4687	4666	4598	4297	4263	4390	3918	3692	3765	3476	3174	3074	2821	2878	2927	2884	2612	2522	2266	2071	2289	2176	1817	1017	223000
		B3	3806	11910	11687	14694	15385	14612	14748	14655	14236	14159	14201	14203	13737	13622	12782	12281	12865	12436	11891	11825	11979	11513	11068	10591	10425	10023	10051	9803	9038	9092	9081	8425	8268	7972	7407	6716	6629	6221	6188	6200	6111	5627	5319	5005	4385	4952	4545	3707	2180	478256
		Total	27540	89088	89848	114054	121569	115615	115354	116354	112199	112561	111738	110323	107603	105942	100237	96851	98782	96861	91981	90595	91074	89289	85626	80567	79947	76856	77186	76190	69679	69530	70213	64422	61981	62058	56361	51868	50267	47208	46725	47131	46903	42539	40559	37294	33507	37669	34450	29048	16514	3697756
Modified protocol	Human	B1	4	6	12	9	9	6	6	8	12	8	9	8	10	9	8	9	13	8	8	10	6	6	3	8	6	7	11	6	2	5	10	5	3	8	8	1	9	7	4	5	3	3	3	4	3	4	4	4	1	321
		B2	65	191	206	223	244	278	224	240	214	231	237	208	220	200	196	214	185	195	203	171	184	170	162	162	176	170	172	151	139	145	167	147	134	119	122	92	113	95	95	102	99	92	99	66	80	74	58	59	42	7631
		B3	3	11	8	12	8	6	8	6	9	8	12	11	10	8	6	6	10	9	10	9	9	12	7	4	9	9	7	8	8	3	7	6	8	6	4	3	6	6	6	3	3	3	2	2	3	6	4	3	1	328
		Total	72	208	226	244	261	290	238	254	235	247	258	227	240	217	210	229	208	212	221	190	199	188	172	174	191	186	190	165	149	153	184	158	145	133	134	96	128	108	105	110	105	98	104	72	86	84	66	66	44	8280
Manufacturer's protocol	*S. aureus*	B1				1			0	0	0				0	0						0		0	0			0	0	0	0			0				0			0			0	0	0						1
		B2				1			0	1	0				0	0						0		0	1			0	1	0	0			1				1			0			0	0	0						6
		B3				2			1	0	0				2	1						1		1	1			1	1	1	3			0				0			0			1	0	1						17
		Total				4			1	1	0				2	1						1		1	2			1	2	1	3			1				1			0			1	0	1						24
Modified protocol	*S. aureus*	B1	0	0	0	0	0	0	0	0	0	0	0	0	0	0	0	0	0	0	0	0	0	0	0	1	0	0	0	0	0	0	0	0	0	0	0	0		0	0	0	0	0	0	0	0	0	0	0		1
		B2	2	5	2	3	5	3	4	5	1	0	2	4	3	2	3	2	1	3	3	3	4	0	3	1	0	3	1	3	1	2	2	0	2	1	3	2		0	0	0	1	1	0	2	0	0	2	0		90
		B3	1	2	7	2	3	5	4	1	0	4	5	4	2	1	4	4	2	4	1	3	3	4	8	5	3	1	5	0	2	1	5	1	2	1	1	3		2	3	2	2	2	2	1	1	1	2	2		124
		Total	3	7	9	5	8	8	8	6	1	4	7	8	5	3	7	6	3	7	4	6	7	4	11	7	3	4	6	3	3	3	7	1	4	2	4	5	0	2	3	2	3	3	2	3	1	1	4	2	0	215
Manufacturer's protocol	Other	B1	32	108	98	153	152	172	157	163	164	171	147	146	146	125	153	114	118	112	116	123	124	117	104	111	118	88	97	104	104	102	90	88	78	63	73	48	63	49	58	43	39	58	40	32	31	34	37	30	11	4704
		B2	4	11	14	19	14	16	13	11	10	18	23	20	14	12	19	11	12	11	13	9	10	10	7	18	16	8	14	13	13	10	13	9	6	7	8	3	9	6	4	2	2	4	5	3	6	2	2	4	4	492
		B3	8	17	14	31	24	39	22	25	26	32	21	33	18	26	25	30	18	17	21	20	26	27	24	14	12	13	19	12	21	21	22	20	17	14	10	15	6	13	9	9	15	7	7	6	10	6	8	5	7	862
		Total	44	136	126	203	190	227	192	199	200	221	191	199	178	163	197	155	148	140	150	152	160	154	135	143	146	109	130	129	138	133	125	117	101	84	91	66	78	68	71	54	56	69	52	41	47	42	47	39	22	6058
Modified protocol	Other	B1	0	0	1	0	1	0	1	0	0	0	0	1	0	0	0	0	1	1	0	0	0	1	1	0	0	1	0	0	0	1	0	1	0	0	0	0	0	0	1	0	0	0	1	0	0	0		0	0	13
		B2	2	3	1	0	0	0	0	1	1	3	1	0	2	4	1	0	0	0	0	0	2	2	0	1	3	1	0	2	0	1	1	0	0	0	0	0	1	2	0	1	2	0	0	1	0	0		1	0	40
		B3	0	1	4	1	0	5	2	1	1	1	3	5	1	3	2	2	1	2	0	2	1	1	0	0	1	0	1	0	3	2	0	2	2	0	2	1	1	1	1	1	0	0	1	0	0	1		0	0	59
		Total	2	4	6	1	1	5	3	2	2	4	4	6	3	7	3	2	2	3	0	2	3	4	1	1	4	2	1	2	3	4	1	3	2	0	2	1	2	3	2	2	2	0	2	1	0	1	0	1	0	112
NTC	Human		2	1				1			1				1																1		1					1							1		1					11
